# ATtRACT—a database of RNA-binding proteins and associated motifs

**DOI:** 10.1093/database/baw035

**Published:** 2016-04-06

**Authors:** Girolamo Giudice, Fátima Sánchez-Cabo, Carlos Torroja, Enrique Lara-Pezzi

**Affiliations:** ^1^Centro Nacional de Investigaciones Cardiovasculares Carlos III, Melchor Fernández Almagro 3, Madrid 28029, Spain; ^2^Bioinformatics Unit, Centro Nacional de Investigaciones Cardiovasculares, Melchor Fernández Almagro 3, Madrid 28029, Spain; ^3^National Heart and Lung Institute, Faculty of Medicine, Imperial College London, London SW7 2AZ, UK

## Abstract

RNA-binding proteins (RBPs) play a crucial role in key cellular processes, including RNA transport, splicing, polyadenylation and stability. Understanding the interaction between RBPs and RNA is key to improve our knowledge of RNA processing, localization and regulation in a global manner. Despite advances in recent years, a unified non-redundant resource that includes information on experimentally validated motifs, RBPs and integrated tools to exploit this information is lacking. Here, we developed a database named ATtRACT (available at http://attract.cnic.es) that compiles information on 370 RBPs and 1583 RBP consensus binding motifs, 192 of which are not present in any other database. To populate ATtRACT we (i) extracted and hand-curated experimentally validated data from CISBP-RNA, SpliceAid–F, RBPDB databases, (ii) integrated and updated the unavailable ASD database and (iii) extracted information from Protein-RNA complexes present in Protein Data Bank database through computational analyses. ATtRACT provides also efficient algorithms to search a specific motif and scan one or more RNA sequences at a time. It also allows discovering *de novo* motifs enriched in a set of related sequences and compare them with the motifs included in the database.

**Database URL:** http:// attract. cnic. es

## Introduction

RNA-binding proteins (RBPs) are key players in several cellular processes. Through their interaction with RNA, RBPs are able to regulate processes such as alternative splicing, transport, localization, stability and translation of RNA (1). RBPs recognize, through particular domains, specific RNA-binding motifs (2). Understanding RBP specificity and identifying their binding motifs is crucial to shed light on the regulatory mechanisms in which they participate. However, the available information on RBPs and their motifs is currently limited, incomplete and sometimes outdated.

Three are the major available sources of data compiling information on RBPs and their binding sites: (i) RBPDB (3) is a repository of experimentally validated RBPs extracted from literature, (ii) CISBP-RNA (4) contains RBPs and binding sites extracted from *in vitro* RNAcompete experiments and a portion of the experimentally validated motifs included in the RBPDB database and (iii) SpliceAid-F (5) is a repository of human splicing factor extracted from literature. Another source of information is the no longer maintained ASD database (6). Among these databases, only RBPDB and SpliceAid-F include a limited number of motifs extracted from protein-RNA complex data buried in Protein Data Bank (PDB) (7). None of the previously described databases integrates a tool for the *de novo* motif analysis, only CISBP-RNA and RBPDB permit to scan sequences for potential binding sites. Moreover, the aforementioned databases are not coherent in terms of reference gene names and/or gene identifier, thus the inconsistency in nomenclature remains a major issue and a source of confusion. For example, the Human CELF1 binding protein is named CUGBP1 in SpliceAid-F as well as in RBPDB, whereas it is named CELF1 in CISBP-RNA. In addition, in RBPDB, CISBP-RNA, SpliceAid-F and ASD it is not possible to identify directly which processes or functions RBPs are involved in.

The goal of our work was: (i) to create a central and coherent repository that collects the RBPs-RNA interactions and RBP-binding motifs extracted from CISBP-RNA, RBPDB, SpliceAid-F,ASD, (ii) to carry out an in silico analysis of the 236 protein-RNA structures available in PDB (release January 2015) and not already assessed by RBPDB and SpliceAid-F, (iii) to reorganize, update and manually curate the last available release of ASD database in order to make the entries again accessible to the public, (iv) to solve the issues of standardization among the existing databases, (v) to integrate Gene Ontology (GO) (7) in order to identify the cellular compartments, the biological processes and the functions in which the RBPs may be involved, (vi) to embed Burrows–Wheeler transform (BWT) algorithm (8) to scan sequences for motifs in a very short time, (vii) to implement MEME (9) and Tomtom (10) programs to permit to the users to analyse their sequences and exploit the information available in the database and (viii) to develop a user-friendly graphical interface that allows users to query the database for a broad type of different requests.

To fulfil these goals, we developed ATtRACT. ATtRACT contains information on 370 hand-curated and experimentally validated RBPs associated with 1583 consensus motifs (Table 1 and the results paragraph for further information) ranging from 4 to 12 nucleotides and belonging to 38 different organisms. On top of that, ATtRACT includes 192 consensus motifs (15%), obtained with the *in* *silico* analysis of protein-RNA complexes extracted from PDB, that are not present in any other database.

**Table 1.** Distribution of motifs extracted from each database included in ATtRACT. The percentage of ATtRACT motifs they represent is shown in brackets.

**Database**	**CISBP-RNA**	**RBPDB**	**SpliceAid-F**	**AEDB**	**PDB**
**Number of consensus motifs**	312 (19.7%)	226 (14.3%)	775 (48.9%)	95 (6.0%)	256 (16.2%)
**Unique motifs**	229 (17.9%)	120 (9.4%)	659 (51.5%)	79 (6.2%)	192 (15.0%)

## Materials and methods

### Implementation

The algorithms are implemented in python 2.7 (https://www.python.org) and C/C ++. SQLite (http://www.sqlite.org) is adopted for organizing and managing the relational database and to store the information. The web2py (http://www.web2py.com) framework and bootstrap (http://getbootstrap.com) framework are used for designing and developing the web interface and for interfacing with the database. Nginx (http://nginx.org) handles the user’s request. The plots are implemented using the D3 library (http://d3js.org) and highcharts library (http://www.highcharts.com). The tables displaying the results are implemented using the javascript plug-in named DataTables (http://www.datatables.net). The logos are generated through WebLogo3 software (http://weblogo.threeplusone.com)

### ATtRACT content

ATtRACT integrates data from RBPDB, CISBP-RNA and SpliceAid-F. For this purpose, we downloaded the available dataset from RBPDB, CISBP-RNA and SpliceAid-F website, and reorganized and completed the missing fields, in order to fit with the database schema of ATtRACT. In order to reduce the proliferation of different gene names and duplicated entries, we changed the RBPs gene names according to UniProt (8) official names. A consistent annotation is essential to create a non-redundant repository and guarantee accurate search results. Furthermore we updated, when available, the gene identifiers according to the last version of Ensembl (9), Xenbase (10) or European Nucleotide Archive (11). We downloaded the ASD data file from ftp://ftp.ebi.ac.uk/pub/databases/astd/aedb/, updated it, and completed the information needed to fill in the fields present in ATtRACT but missing in the ASD data file. Each entry in our database corresponds to a RBP and to its associated binding sites. Each record is annotated with the official gene name and its synonyms, the gene identifier, the motif associated to the RBP, the type of experiment performed for the detection of the motif, PubMed identifier and the domains associated to the RBP according to PFAM (12) or InterPro (13) annotations. Additionally, we extracted, through Ensembl Biomart services (14), GO annotations associated to each RBP and integrated them in ATtRACT. Finally, for each motif, we added the sequence logo and quality score.

### Quality score

Quality score estimates the binding affinity between RBPs and binding sites. Experiments such as systematic evolution of ligands by exponential enrichment (SELEX) permit to identify winner sequences, i.e. sequences with a strong preference to bind to RBPs. The functional motif is assessed through the alignment of winner sequences. The result of the alignment is often represented through IUPAC ambiguous notation (15) and shows the most frequent nucleotides found at each position. This representation has clear limitations because each position is not evaluated quantitatively (16). In AEDB and SpliceAid-F databases the motifs assessed by SELEX or RNAcompete experiments are considered equally likely, therefore it is not possible to evaluate the binding affinity between RBPs and motifs. To solve this problem, we manually extracted from the literature the winner sequences and aligned them in order to represent the binding preference through a position-specific probability matrix (PPM). If the winner sequences are not annotated, the PPM is generated considering the IUPAC letter encoding for more than onenucleotide as equally likely.

Formally the score *S* of a matrix *M* for a motif *m* of length *l* is defined as:

$$S=\;\prod_{i=1}^{l}P\left(m_{i}||M\right)$$

where *P*$\left(m_{i}||M\right)$ is the probability of observing the nucleotide *m* in position *l* in the PPM matrix. The quality score represents the probability of observing a given motif within the experiment. Note that each motif in ATtRACT is associated to a PPM. For this reason and according to the definition of quality score, the motifs coming from single sequence experiments, such as UV cross-linking or Electrophoretic Mobility Shift Assay (EMSA), have a quality score equal to 1.0, since it is possible to assess only one motif in the experiment.

### PDB data

ATtRACT is completed with motifs extracted from RBP-RNA interactions based on structural data. PDB is a repository of 3D structures of proteins and molecular complexes.

Instead, to take advantage of the information present in PDB, we developed a pipeline to obtain RNA sequence motifs from the structural information stored in PDB experiments.

A total of 236 proteins structures, excluding the ribosomes, were downloaded from PDB (release January 2015). All structures belong to Eukaryotes and contain at least a complex of protein and RNA. The experimental methods used for the detection of protein-RNA contacts were Nuclear Magnetic Resonance (NMR), electron microscopy and X-ray crystallography with a resolution better than 3.9 Å. Two types of bond interactions, between RPBs-RNA complexes, are taken into consideration: the van der Walls forces and the hydrogen bond. The HBPLUS (17) program was used to assign H-Bond in X-ray crystallographic data. The NUCPLOT (18) program was used to identify proteins-nucleic acid contacts based on a distance criterion. We set the distance cut-off for hydrogen bonds to 3.0 Å and the distance cut-off for van der Waals bonds to 3.9 Å. We considered a binding site if four or more contiguous nucleotides satisfied the distance criterion defined previously and interacted with any atom of the protein. To confirm the reliability of our method, we performed an ungapped alignment between the motifs extracted from PDB and the motifs belonging to the same RBP and verified by another type of experiment. We were able to align 93 motifs out of 256, belonging to 43 RBP. Forty-eight motifs (51.6%) are perfectly aligned, 27 (29%) differ only in one nucleotide, 4 (4.3%) differ in two nucleotides, the remaining 14 motifs (15.1% of the total) differ in more than two nucleotides. For the latter we provide evidence from the literature demonstrating that 13 motifs out of 14 are correct even if they are completely different from the motif confirmed with another type of experiment (Supplementary Figure S1).

### Sequence scan

In ATtRACT, we implemented the BWT algorithm to find those motifs that perfectly match a given sequence or set of sequences (FASTA or multi-FASTA file of 20 000 nucleotides maximum). The BWT currently represents one of the most efficient algorithms; it permits to scan, in a very short time, the input sequences and to locate the positions of any or of a subset of motifs present in ATtRACT through a perfect match comparison. Therefore, the algorithm reports a hit only if the motif and the k-mers extracted from the sequence are equal. Additionally, given the high number of motifs available in ATtRACT and because of their length, it is very easy to find many of those motifs in any input sequence provided. In order to assess the possible biological relevance, we provide log-odds scores. Three log-odds scores are assigned to each motif of the following species: *Caenorhabditis elegans*, *Drosophila melanogaster*, *Homo sapiens*, *Mus musculus*, *Saccharomyces cerevisiae* and *Xenopus tropicalis* since they are the most characterized species in ATtRACT. To better understand how the log odd score is calculated, we introduce the concept of genomic functional context (GFC). A genomic functional context is a collection of three distinct dataset each one belonging to a species specific genomic region. Each datasets, in fact, contains, respectively, the sequences of: (i) all the exons plus 250 nucleotides upstream and downstream (ii) all the introns (iii) all the coding sequences. The log odd score is defined as the ratio between the probability of locating the motif in the input sequence divided by the probability of finding the same motif in any of the genomic functional context of the reference species. Therefore a log odd score >0 means that the probability of finding the motif is greater in the input sequence with respect to the corresponding genomic functional context and vice versa if <0.

Formally, the score is calculated in the following way:

Let *M *= [*m*_1_, *m_2_*_,_ *m*_3_… *m_n_*] be the set of the motifs present in the database.

We define GFC = [exon ± 250, intron, Coding DNA Sequence (coding sequence) (CDS)] as the collection of distinct datasets containing, respectively, the sequences belonging to all exons plus 250 nucleotides upstream and downstream, all introns and all coding sequences of the reference organism.

*S ^[GFC]^* =* *[*s*_1_, *s*_2_ … *s*_n_] where *s*_1_, *s*_2_ … *s_n_* are the sequences in the reference organism that represent the genomic functional context. That is, *S* ^[intron_human]^ represents all introns in human

*C^S[GFC] ^= *[*c_m1_*,*c_m2_*,*c_m3_*,…,*c_mn_*] where *c_m1_*,*c_m2_*,*c_m3_*,…,* c_mn_* are the occurrences of motifs *m_1_*,*m_2_*,*m_3_*,…,*m_n_* in *S^[GFC]^*

Let *s be* an input sequence of length *l_s_* and *m_x_* ∈ *M* a motif of length *l_m_* of multiplicity *t* found in the input sequence. The log odd ratio is defined as:

$${OR}_{S[GFC]}=\;\log_{2}\frac{Obs}{{Exp}_{S[GFC]}}$$

where *Obs* is defined as:

$$Obs=\;\frac{t}{l_{s}-\;l_{m}+\;1}$$

$${Exp}_{s[GFC]}=\;\frac{c_{mx}}{\sum_{i=1}^{n}\;[len\;\left(s_{i}^{\left[GFC\right]}\right)-\;{l\;}_{m}+1]}$$

Where $len\;\left(s_{i}^{\left[GFC\right]}\right)$ is the length of the *i^th^* sequence in *S^[GFC]^*.

### MEME and Tomtom

In ATtRACT, we integrated MEME and Tomtom programs in order to allow users to discover motifs that occur frequently in a set of sequences and/or compare them with the ones present in the ATtRACT database. MEME takes advantages of an extension of the Expectation Maximization algorithm to produce a statistical model that permits to find a relationship between possibly related unaligned sequences. Tomtom allows evaluating whether a *de novo* motif, enriched in a set of sequences, looks like to any other motif present in the ATtRACT database. Tomtom assigns a score, for each *de novo* motif, based on the expected value. The E-value describes the number of hits one can expect by chance in a database of a particular size. The closer to zero the E-value, the more plausible the match is. Since the E-value depends on the database’s size, ATtRACT permits to extract a subset of the database according to the length of the motif, the experiment assessed, the organism and the domain, in order to refine the search and increase the E-value.

## Results

**Figure 1.**
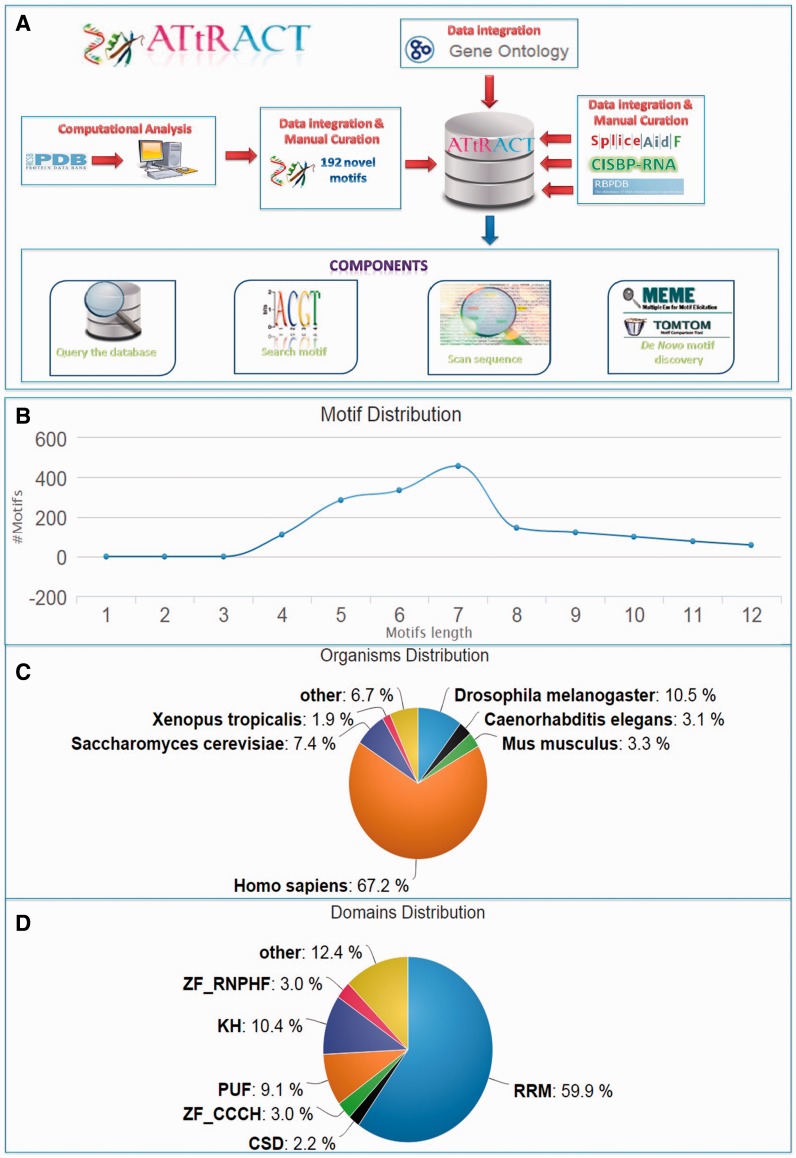
ATtRACT database. (**A**) Data flows in ATtRACT database. (**B**) Distribution of motif length in the ATtRACT database. (**C**) Organism distribution for all the different RNA motifs included in the database. (**D**) Frequency distribution of the different protein domains in the RBPs included in the ATtRACT database.

ATtRACT contains information on 370 hand-curated and experimentally validated RBPs associated with 1583 consensus motifs out of which 192 are not present in any other database and they account for the 15% of the total content of ATtRACT database. Table 1 shows the motifs distribution extracted from each database included in ATtRACT and the percentage of ATtRACT motifs that they represent. Note that the table takes into consideration the binding specificity of the RBP, the experiments and the organisms, meaning that a motif is considered a distinct entry if: (i) it binds to a different RBP or (ii) it was identified with a different experimental approach or (iii) it was identified in a different organism. For example, the motif ‘ACGCGCC’ is considered as two distinct entries because it binds either SRSF1 or RBM8A. The motifs length range from 4 to 12 nucleotides (Figure 1B). The database compiles motifs and RBPs from 38 different organisms (Figure 1C). Among the RBPs included in ATtRACT, the RNA recognition motif is the most represented domain (Figure 1D). Four are the main components of the ATtRACT database. The first permits to query the database. The second searches for a specific motif. The third scans one or more RNA sequences searching for RBP-binding sites. The fourth allows discovering enriched motifs in a set of related sequences and comparing them with the motifs present in ATtRACT. In addition, ATtRACT permits to download the entire database and the results provided to the users.

### Search for RBPs

The search interface offers a broad range of possibilities. Users can search information about specific entries of the database simply by typing or choosing one or a combination of the following options: Official Gene name (i.e. ‘SRSF1’), Synonyms (i.e. ‘SFRS1’) Gene ID (i.e. ‘ENSG00000136450’), minimum or maximum length of motifs, type of experiments, organisms and/or domains. Search criteria can be combined by using combinations of queries.

**Figure 2.**
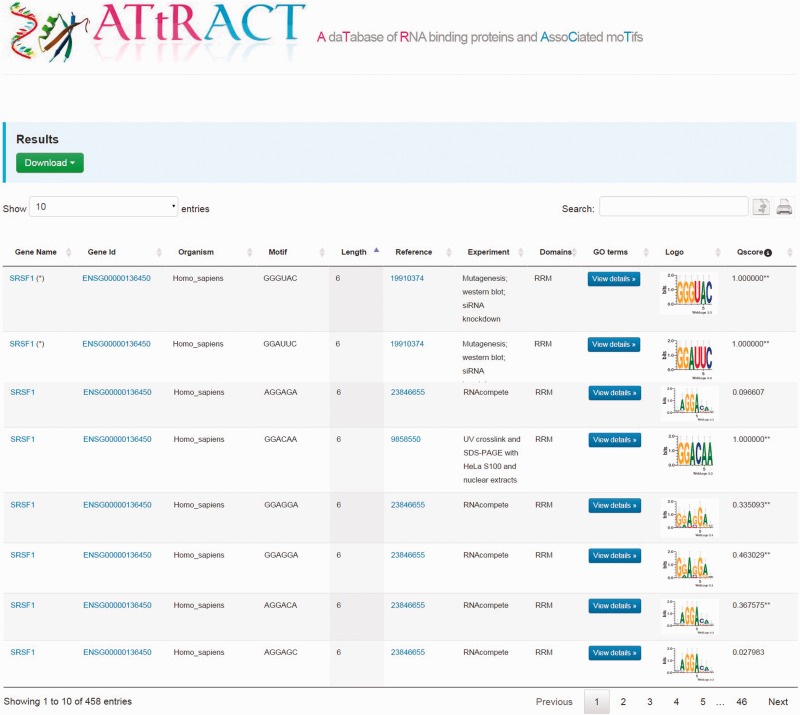
Example of the ATtRACT search interface showing the search result for SRSF1. Users can download the results, search inside the table using the search input box, print the table and copy the table to the clipboard. Moreover, it is possible to order the table clicking on the header and explore the annotated GO terms with which the RBPs are associated.

The results are displayed as tables (Figure 2). A file, containing the search results, can be downloaded by clicking on the drop-down menu on the top of the page and choosing the preferred format between commas or tab separated value. The users can further filter the entries of the table through a whole text search using the search box. The users can also copy results to the clipboard or print them. The tables are sortable simply by clicking on the header. The table headers display the following fields: gene name, gene identifier, organism, binding sites, PubMed identifier, type of experiment, domain, GO, the sequence logo and quality score. It is possible to investigate the GO terms associated to RBPs by clicking the corresponding button in the GO terms column. A popup window will show the GO terms associated to RBP. A hyperlink in the cell table redirects the user to UniProt database when clicking on gene name, to the Ensembl database when clicking on gene identifier. In few cases, the redirection, due to the lack of annotation, occurs through other repositories. Moreover, when the experiment is NMR or X-ray we have added a hyperlink to the corresponding PDB entry in the column experiment. Finally, clicking on motif logo is possible to download the PPM.

### Search for a specific motif

Users can search for specific motifs by submitting a sequence ranging from 4 to 12 nucleotides. The search engine supports IUPAC ambiguous notation. By default, the search engine retrieves any motif that contains the sequence in the input. The user can execute a perfect match search simply by enclosing the input sequence between quotes (see web for examples). The results are displayed as a table and are downloadable. The result table shows the same fields mentioned in the previous paragraph (see search for RBPs paragraph and Figure 1).

### Scan sequence interface

**Figure 3.**
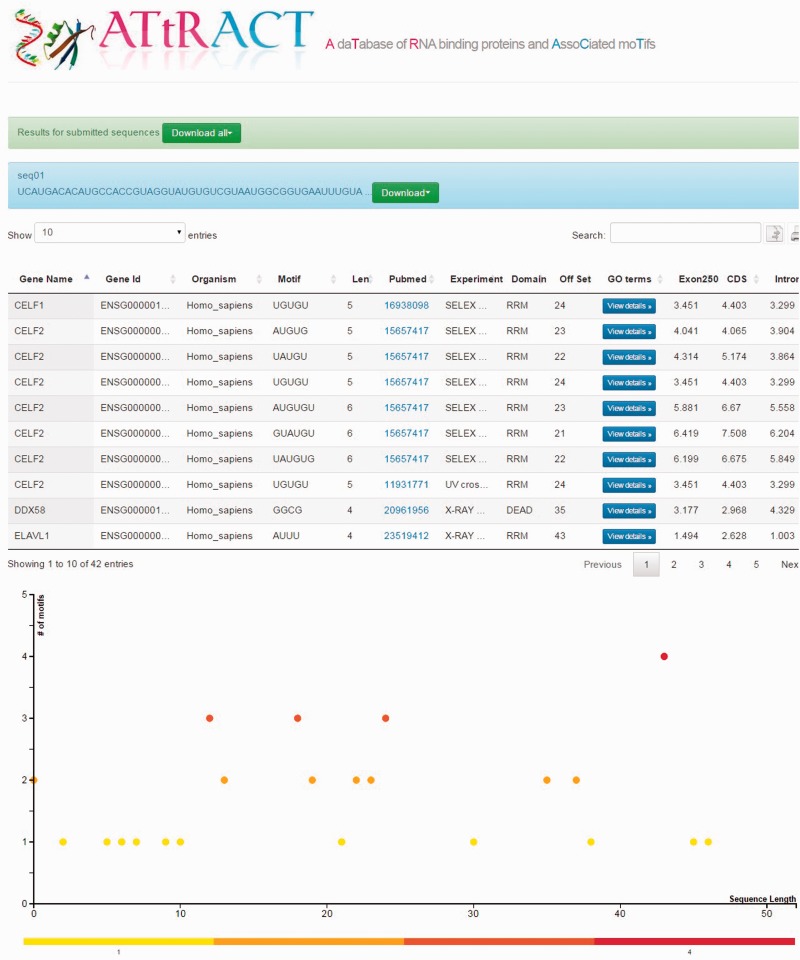
Example of the ATtRACT result page showing the sequence scan for an input sequence. On the top of the page the results appear in table format. The bottom of the page displays the graph showing the frequency and position of motifs in the input sequence.

Users can upload a file containing one or more RNA/DNA sequences in fasta or multi-fasta format and scan the file searching for the presence of motifs. The user can restrict the search by selecting a specific organism and/or motifs of a certain length. The results are provided in a tabular format (Figure 3) and are downloadable. Four more fields are added to the headers of the results tables: (i) the offset (ii) Exon250, (iii) CDS and (iv) intron. The offset represents the distance in terms of nucleotides at which it is possible to locate the motif, starting from the beginning of the sequence. The other fields represent the log odd ratio of the selected motif in each of the indicated regions (see Materials and methods). ATtRACT provides a plot for each scanned sequence, in order to better visualize the results. The *x*-axis represents the sequence length; each bin represents a nucleotide of the input sequence. The *y*-axis represents the amount of motifs. Each point represents the starting position of a motif across the input sequence. The higher the dot is on the *y*-axis, the more motifs start in that position. A red-yellow colour scale distinguishes the dots. The redder the dots are, the higher the concentration of motifs is. It is possible to zoom-in the graph with the mouse wheel and interact with it by clicking on the dots. A table will show all RBPs, the motifs and the organisms associated with that position.

### MEME and Tomtom interface

With *de novo* motif discovery, users can: (i) upload a set of sequences in multi-fasta format (3000 nucleotides maximum). The sequences are analysed by MEME in order to find *de novo* motifs or (ii) upload the output of a *de novo* motif analysis done through MEME/MEMERIS (19) or (iii) upload a position weight matrix (PWM) representing the results of other *de novo* motif finder such as DRIMust (20), XXmotifs (21) or cERMIT (22). Then each *de novo* motif is analysed by Tomtom in order to find whether a *de novo* motif looks like any other motif present in ATtRACT. If the user decides to submit a multi-fasta file, other parameters must be taken into consideration. The motif distribution indicates how the occurrences of motifs are distributed along the sequences. Three possibilities are available (i) one motif per sequence (ii) zero or one motif per sequence (iii) any number of repetitions (for further information visit: http://meme.nbcr.net/meme/meme-input.html).The field named E-value indicates a significance threshold for both MEME and Tomtom. Users can also choose whether they want to extract a subset of the ATtRACT database in order to improve the E-value. The results page is divided into two sections: the *de novo* motifs discovered by MEME/MEMERIS or uploaded by the users through a PWM are shown at the top of the page and the significant matches discovered by Tomtom follow. The Tomtom results table is subdivided into three columns: the first represents the *de novo* motifs, the second represents a brief summary of the features of the RBP and the third provides an alignment figure between the motif present in the ATtRACT database and the *de novo* motif (see Supplementary Figure 2). Both the results of MEME analysis (if performed through our database) and the significant matches discovered by Tomtom are downloadable.

## Conclusion

ATtRACT collects in a unique resource all available information about RBPs and their associated motifs. In comparison to other similar databases, ATtRACT adds 192 motifs not present in any other database from 110 different RBPs by retrieving the information buried in the PDB database In addition, ATtRACT permits to investigate the GO terms associated to the RBPs. To our knowledge, ATtRACT is the largest and most updated collection of RBPs and associated binding sites. For this reason, it represents an invaluable resource to improve our understanding of protein-RNA interactions and how they are regulated. ATtRACT, thanks to the presence of experimentally validated data, could be useful for the development of new and more accurate machine learning methods for the prediction of RBP-binding sites. Finally, ATtRACT will be constantly updated with the new releases of PDB data.

## Supplementary Material

Supplementary Data
