# Supplementary material for: ATtRACT—a database of RNA-binding proteins and associated motifs
Source: Database (Oxford). 2016 Apr 6;2016:baw035. doi: 10.1093/database/baw035 (PMC4823821; doi:10.1093/database/baw035)
Supplement: Supplementary Data [file supp_2016_baw035_index.html]

Supplementary Data 

# ATtRACT—a database of RNA-binding proteins and associated motifs

## Supplementary Data

files

- Supplementary Data - pdf file
- Supplementary Data - pdf file
