# Supplementary material for: ATtRACT—a database of RNA-binding proteins and associated motifs
Source: Database (Oxford). 2016 Apr 6;2016:baw035. doi: 10.1093/database/baw035 (PMC4823821; doi:10.1093/database/baw035)
Supplement: Supplementary Data [file supp_baw035_supplementary_figure_1.pdf]

>ENSG00000149187
UUGUG
||||||
UGUGUG
distance: 1

>ENSG00000149187
UUGUU
|||||
UGUGU
distance: 2

>ENSG00000149187
UGUU
|||||
UGUGU
distance: 1

>ENSG00000149187
UGUGUG
||||||
UGUGUG
distance: 0

>ENSG00000149187
UGUUU
|||||
UGUGU
distance: 1

>ENSG00000161547
UCCAGU
|||||||
UCGAGAU
distance: 1

>ENSG00000161547
UGGAGU
|||||||
UGGAGAU
distance: 1

>ENSG00000011304
UCUCU
|||||
UCUCU
distance: 0

>ENSG00000011304
CUCUCU
||||||
CUCUCU
distance: 0

```
>ENSG00000011304
CUCU
||||
CUCU
distance: 0

>ENSG00000197111
CCCUAA
||||||||
CCCUUAAA
distance: 1

>ENSG00000197111
CCCU
||||||
CCAUUC
distance: 1

>FBGN0264270 see Hennig,J., Militti,C., Popowicz,G.M., Wang,I.,
Sonntag,M., Geerlof,A., Gabel,F., Gebauer,F. and Sattler,M. (2014)
Structural basis for the assembly of the Sxl-Unr translation regulatory
complex. Nature, 515, 287-290. (see figure 2f )
GUGA
|||||||
UUUUUUU
distance: 6

>FBGN0264270
UGUUUUUUUU
  ||||||||
  UUUUUUUU
distance: 0

>FBGN0264270 see Hennig,J., Militti,C., Popowicz,G.M., Wang,I.,
Sonntag,M., Geerlof,A., Gabel,F., Gebauer,F. and Sattler,M. (2014)
Structural basis for the assembly of the Sxl-Unr translation regulatory
complex. Nature, 515, 287-290.
GCACG
|||||||
UUUUUUU
distance: 7

>FBGN0264270
UUUUUUUGAGCA
|||||||
UUUUUUU
distance: 0

>ENSG00000063244
UUUUUUU
|||||||
UUUUUUC
distance: 1
```

```
>ENSG00000063244
UUUUU
|||||||
UUUUUCC
distance: 0

>ENSG00000234414
 ACAAGAC
|||||
CACAA
distance: 1

>ENSG00000131914
AGGAGAU
|||||||
AGGAGAA
distance: 1
>ENSG00000138385
     UUUU
|||||||||
UGCUGUUUU
distance: 0

>ENSG00000138385
    AUUU
|||||||||
UGCUGUUUU
distance: 1

>ENSG00000138385
UGCUG
|||||||||
UGCUGUUUU
distance: 0

>WBGENE00001595
  CUAAC
||||||||||||
AUCUACUCAUAU
distance: 2

>WBGENE00001595
  CUACUCAUAU
||||||||||||
AUCUACUCAUAU
distance: 0

>ENSG00000078328
UGCAUGU
||||||
UGCAUG
distance: 0
```

>YOR359W see Aviv,T., Lin,Z., Ben-Ari,G., Smibert,C.A. and Sicheri,F. (2006) Sequence-specific recognition of RNA hairpins by the SAM domain of Vts1p. Nat. Struct. Mol. Biol., 13, 168–176. (figure 3a)
UCUUUGA
|||||||
GCUGGUG
distance: 4

>YOR359W
 CUGGCA
|||||||
GCUGGCC
distance: 1

>XB-GENE-6252591 see Lee,B.M., Xu,J., Clarkson,B.K., Martinez-Yamout,M.A., Dyson,H.J., Case,D.A., Gottesfeld,J.M. and Wright,P.E. (2006) Induced Fit and 'Lock and Key' Recognition of 5 S {RNA} by Zinc Fingers of Transcription Factor {IIIA}. J. Mol. Biol., 357, 275–291. (figure 1b left)
 CCUGGUUAG
|||||||
GGGUGGG
distance: 5

>XB-GENE-6252591 see Lee,B.M., Xu,J., Clarkson,B.K., Martinez-Yamout,M.A., Dyson,H.J., Case,D.A., Gottesfeld,J.M. and Wright,P.E. (2006) Induced Fit and 'Lock and Key' Recognition of 5 S {RNA} by Zinc Fingers of Transcription Factor {IIIA}. J. Mol. Biol., 357, 275–291. (figure 1b right)
CCAUAC
|||||||
GGGUGGG
distance: 6

>ENSG00000147274
 UCAAA
||||||
AUCAAA
distance: 0

>ENSG00000134644
UGUAAUAUU
||||||||
UGUAAAUA
distance: 1
>ENSG00000134644 see Gupta,Y.K., Nair,D.T., Wharton,R.P. and Aggarwal,A.K. (2008) Structures of Human Pumilio with Noncognate {RNAs} Reveal Molecular Mechanisms for Binding Promiscuity. Structure, 16, 549-557.
UUUAAUGUU
||||||||
UGUAAAUA
distance: 4

```
>ENSG00000134644
UGUAUAUA
||||||||
UGUAUAUA
distance: 0

>ENSG00000134644
UGUAAAUA
||||||||
UGUAAAUA
distance: 0

>ENSG00000134644
UGUACAUA
||||||||
UGUACAUA
distance: 0

>ENSG00000134644
UGUACAUC
||||||||
UGUACAUA
distance: 1

>ENSG00000134644
UGUAGAUA
||||||||
UGUAAAUA
distance: 1

>ENSG00000134644
UGUCCAG
||||||||
UGUACAUA
distance: 2

>ENSG00000134644
UGUACAU
||||||||
UGUACAUA
distance: 0

>ENSG00000134644
UGUAUAU
||||||||
UGUAUAUA
distance: 0

>ENSG00000066044
AUUUU
|||||
AUUUA
distance: 1
```

```
>ENSG00000066044
UUUU
|||||
UUUUU
distance: 0

>ENSG00000066044
AUUU
|||||
AUUUA
distance: 0

>ENSG00000112531
ACUAAC
||||||
ACUAAC
distance: 0

>YGL014W
UGUAU
||||||||
UGUAUAUA
distance: 0

>YGL014W
UGUAUAUUA
|||||||||
UGUAUAUUA
distance: 0

>YGL014W
UGUAUAUA
||||||||
UGUAUAUA
distance: 0

>ENSG00000070756
AAAAAAAA
|||||||
AAAAAAA
distance: 0

>ENSG00000070756
AAAAAAA
|||||||
AAAAAAA
distance: 0

>ENSG00000077312
AUUGCACC
|||||||
AUUGCAC
distance: 0
```

```
>ENSG00000077312
AUUGCAC
|||||||
AUUGCAC
distance: 0

>ENSG00000102081 no reference
GCUGC
|||||||
GGACAGG
distance: 4

>YGL044C see Leeper,T.C., Qu,X., Lu,C., Moore,C. and Varani,G. (2010)
Novel Protein–Protein Contacts Facilitate mRNA 3′-Processing Signal
Recognition by Rna15 and Hrp1. J. Mol. Biol., 401, 334–349.
AAUAAU
||||||
UGUUGU
distance: 4

>YGL044C see Leeper,T.C., Qu,X., Lu,C., Moore,C. and Varani,G. (2010)
Novel Protein–Protein Contacts Facilitate mRNA 3′-Processing Signal
Recognition by Rna15 and Hrp1. J. Mol. Biol., 401, 334–349.
UAUAUAUAA
||||||
UGUUGU
distance: 5

>ENSG00000162374
 UAUUUAUUUA
||||||||||
UUAUUUAUUU
distance: 1

>ENSG00000162374
AUUU
 |||||||
 UUUUUUU
distance: 1

>ENSG00000113742
CUUUA
||||||
UUUUUU
distance: 2

>ENSG00000055917
UGUACAUC
||||||||
UGUACAUA
distance: 1
```

```
>ENSG00000055917
UGUAGAUA
||||||||
UGUAGAUA
distance: 0

>ENSG00000055917
UGUAAAUA
||||||||
UGUAAAUA
distance: 0

>YLL013C
  UGUAUAUA
||||||||||
CAUGUAUAUA
distance: 0

>YLL013C
  UGUAAAUA
||||||||||
CAUGUAAAUA
distance: 0

>YOL123W
UAUAUAU
|||||||
UAUAUAA
distance: 1

>ENSG00000139910
 UCACC
||||||
AUCACC
distance: 0

>ENSG00000139910
 CAGUCAC
||||||||
UCAGUCAC
distance: 1

>WBGENE00011279 see Kuwasako,K., Takahashi,M., Unzai,S., Tsuda,K.,
Yoshikawa,S., He,F., Kobayashi,N., Guntert,P., Shirouzu,M., Ito,T., et
al. (2014) RBFOX and SUP-12 sandwich a G base to cooperatively regulate
tissue-specific splicing. Nat. Struct. Mol. Biol., 21, 778-786. (results
and figure 1a)
 GUGUGC
|||||||
AGCAUGC
distance: 3
```

```
>WBGENE00011279
UGCAUGG
|||||||
UGCAUGA
distance: 1

>ENSG00000104967 see Lewis,H.A., Musunuru,K., Jensen,K.B., Edo,C.,
Chen,H., Darnell,R.B. and Burley,S.K. (2000) Sequence-Specific {RNA}
Binding by a Nova {KH} Domain: Implications for Paraneoplastic Disease
and the Fragile X Syndrome. Cell, 100, 323-332. (figure 2 complex 1)
CCUAGAUCACC
||||||
AACACC
distance: 5

>ENSG00000104967
GAUCACC
 ||||||
 AUCACC
distance: 0

>ENSG00000152518
UUAUUUAUU
|||||||||
UUAUUUAUU
distance: 0

>ENSG00000120948
GUGAAUGA
  ||||||
  GAAUGA
distance: 0

>WBGENE00001402
UGUGUUAUC
|||||||||
UGUGUUAUC
distance: 0

>WBGENE00001402
UGUGCCUUA
|||||||||
UGUGCCAUA
distance: 1

>WBGENE00001402
UGUAA
|||||||||
UGUAAAAUC
distance: 0
```

>WBGENE00001402
UGUACCAUA
|||||||||
UGUACCAUA
distance: 0

>WBGENE00001402 see Qiu,C., Kershner,A., Wang,Y., Holley,C.P., Wilinski,D., Keles,S., Kimble,J., Wickens,M. and Hall,T.M.T. (2012) Divergence of Pumilio/fem-3 mRNA binding factor (PUF) protein specificity through variations in an RNA-binding pocket. J. Biol. Chem., 287, 6949-6957. (see paragraph An Upstream C Is Required for Tight Binding by FBF)
CAUGUGC
|||||||||
  UGUGUCAUC
distance: 5

>WBGENE00001402 see Wang,Y., Opperman,L., Wickens,M. and Hall,T.M.T. (2009) Structural basis for specific recognition of multiple mRNA targets by a PUF regulatory protein. Proc. Natl. Acad. Sci. U. S. A., 106, 20186-20191. (figure 2a)
CUGUGC
|||||||||
 UGUGCCAUA
distance: 1

>WBGENE00001402 see Wang,Y., Opperman,L., Wickens,M. and Hall,T.M.T. (2009) Structural basis for specific recognition of multiple mRNA targets by a PUF regulatory protein. Proc. Natl. Acad. Sci. U. S. A., 106, 20186-20191. (figure 2b)
 AUAC
|||||||||
UGUAAAAUC
distance: 3

>WBGENE00001402
UGUGUCAUU
|||||||||
UGUGUCAUU
distance: 0

>WBGENE00001402
UGUGC
|||||||||
UGUGCCAUA
distance: 0

>WBGENE00001402
UGUACUAUA
|||||||||
UGUACUAUA
distance: 0

```
>ENSG00000048740
 UGUU
|||||
AUGUU
distance: 0

>ENSG00000168066
AUACUAACAA
|||||||||||
UAUACUAACAA
distance: 0

>ENSMUSG00000003410
AUUUAUUUU
 |||||||
 UUUUUUU
distance: 1

>ENSG00000136527
 AGAA
|||||
AAGAA
distance: 0

>ENSG00000136527
 AGAAC
||||||
AAGAAC
distance: 0

>ENSG00000136450
UGAAGGAC
||||||||
AGAAGGAC
distance: 1

>WBGENE00006321
 GUGUGC
|||||||
AGUGUGA
distance: 1

>WBGENE00006321
 GUGUG
|||||||
AGUGUGA
distance: 0

>YLR116W
AUACUAAC
 |||||||
 UACUAAC
distance: 0
```

```
>YLR116W
UACUAACA
|||||||
UACUAAC
distance: 0

>YLR116W
UACUAAC
|||||||
UACUAAC
distance: 0
```
