# Supplementary material for: ATtRACT—a database of RNA-binding proteins and associated motifs
Source: Database (Oxford). 2016 Apr 6;2016:baw035. doi: 10.1093/database/baw035 (PMC4823821; doi:10.1093/database/baw035)
Supplement: Supplementary Data [file supp_baw035_Supplementary_figure_2.pdf]

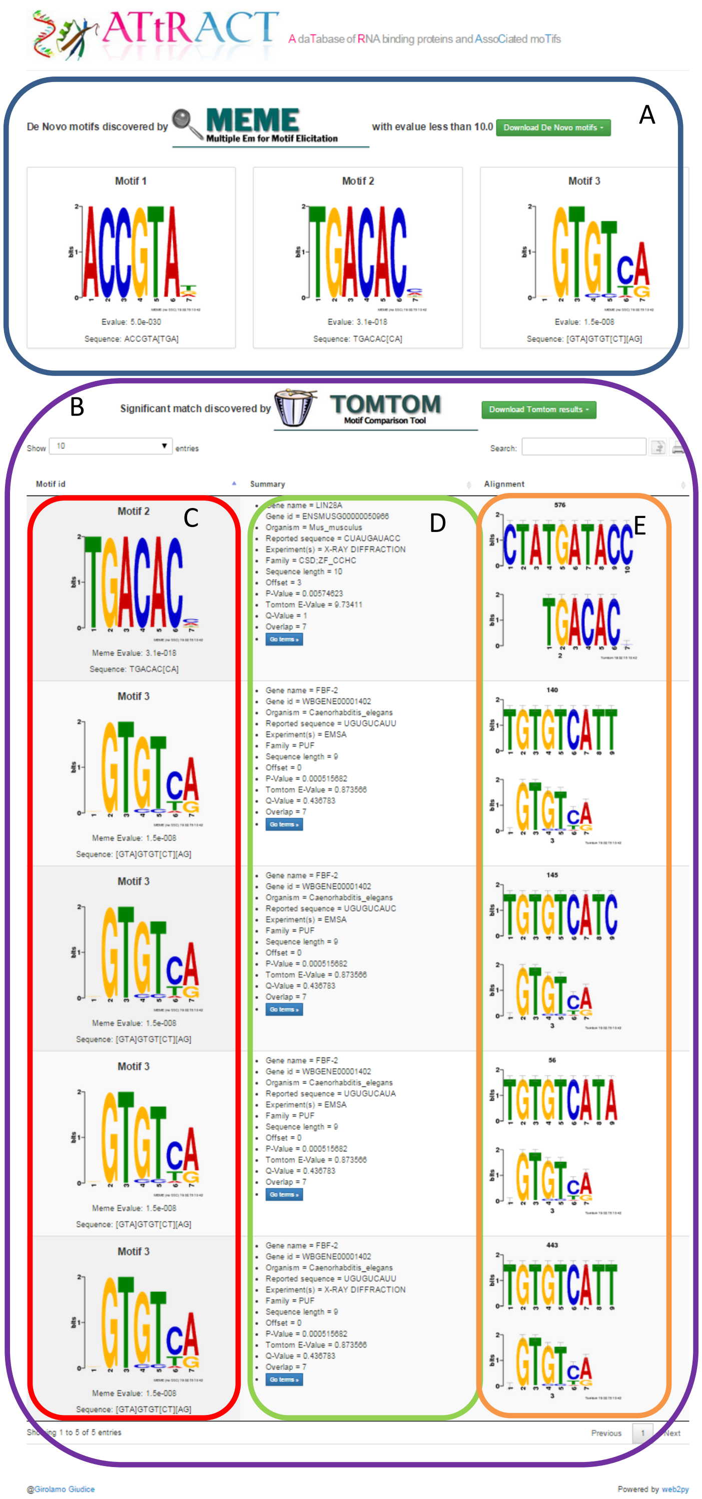

Supplementary Figure 1. Typical output of De Novo motif analysis. A) De Novo motifs discovered by MEME. B) Tomtom Output. C) Enriched motifs discovered by MEME. D) Brief summary of the characteristics of the RBPs that bind the motif. E) Alignment between the motifs present in the ATtRACT and the ones discovered by MEME.
